# Follow-up survey of Japanese medical students’ interactions with the pharmaceutical industry

**DOI:** 10.1371/journal.pone.0206543

**Published:** 2018-11-02

**Authors:** Sayaka Saito, Takami Maeno, Yasushi Miyata, Tetsuhiro Maeno

**Affiliations:** 1 Department of Primary Care and Medical Education, University of Tsukuba, Tsukuba, Ibaraki, Japan; 2 Department of Primary Care and Community Health, Medical Education Center, Aichi Medical University School of Medicine, Nagakute, Aichi, Japan; University of Newcastle, AUSTRALIA

## Abstract

Interaction of medical students with the pharmaceutical industry is common. However, students are thought to be vulnerable to the influence of this interaction, and regulations to limit such interactions are required. The Japan Pharmaceutical Manufacturers Association revised its promotion code in 2013 and specified upper limits for promotional aids. We aimed to investigate whether Japanese medical students’ interactions with the pharmaceutical industry changed from 2012 to 2016. This study solicited the participation of all medical schools in Japan. An anonymous cross-sectional survey was administered to medical students from May 2016 to March 2017 to investigate their interactions with the pharmaceutical industry. The results were compared with those of a previous study conducted in 2012. Forty of the 80 medical schools in Japan participated. The student response rate was 74.1%, with 6771 (3395 preclinical, 3376 clinical) evaluable responses. More than 98% of clinical students had previously accepted stationery, a brochure, or a lunch, and significantly higher percentages of clinical students had accepted these items in 2016 than in 2012 (*p* < .001). The interactions between clinical students and pharmaceutical companies increased slightly between 2012 and 2016. This study will hopefully promote discussion regarding the regulation of student–industry interactions.

## Introduction

Studies in many countries have shown that the pharmaceutical industry frequently interacts not only with physicians, but also with medical students [1–5]. The physician–industry relationship can lead to lower prescribing quality [6,7]. Trainees and students are thought to also be vulnerable to the influence of the pharmaceutical industry, and regulations to limit interactions between students and the industry are required [8,9].

Attempts to reduce industry influence include both government regulation and self-regulation [10]. An example of government regulation is the Physician Payment Sunshine Act, proposed in 2009 in the United States to require pharmaceutical companies to disclose payments and gifts to physicians [11]. In Finland, legislative reform passed in 2004 instituted fines for both physicians and the pharmaceutical industry in cases where gifts have more than a modest monetary value and where gifts or events have no educational value [12]. However, in Japan, there are no laws regulating or requiring disclosure of the relationship between physicians and the pharmaceutical industry.

Self-regulatory measures have been published by both the pharmaceutical industry and professional medical organizations in the United States. In 2008, the American Medical Association and the American College of Physicians released stringent ethical codes, and the Association of American Medical Colleges released a task force report calling on academic health centers to develop rules regarding interactions with the industry in the same year [8]. The Institute of Medicine has also released a recommendation report on conflicts of interest [13], and the Pharmaceutical Research and Manufacturers of America revised their promotion code in 2009 [14]. Only a few studies have followed up the frequency of interactions between medical students and the pharmaceutical industry as a result of these changes [5,12]. One follow-up survey in the United States reported that the number of interactions of any kind between medical students and the pharmaceutical industry decreased from 4.1 per month in 2003 to 1.6 per month in 2012, and the percentage of medical students who received any gifts from industry representatives decreased from 100% to 79% over the same period [5].

In Japan, professional societies have not published recommendations or restrictions on the physician–industry relationship, although a 2008 recommendation by the Association of American Medical Colleges being translated into Japanese in 2012. The Japan Pharmaceutical Manufacturers Association (JPMA) revised its promotion code in 2013 to meet the International Federation of Pharmaceutical Manufacturers & Associations’ Code of Practices, revised in 2012, which specified that promotional aids should cost 3,000 yen (approximately US$30) or less and that items of medical utility provided to physicians should be less than 3,000 to 5,000 yen (approximately US$30 to US$50) in value [15]. A national survey of Japanese medical students conducted in 2012 reported that 98% of clinical students had received stationery such as pens and notepads and that 97% had received a lunch at a promotional meeting for pharmaceutical industry products [16]. However, the interactions of Japanese medical students with the pharmaceutical industry have not been evaluated since 2012.

We aimed to examine whether there were any changes in Japanese medical students’ interactions with the pharmaceutical industry between 2012 and 2016.

## Methods

The questionnaires administered to students were part of a larger, anonymous, self-administered survey investigating medical students’ interactions with and attitudes toward the pharmaceutical industry. The institutional review board at Tsukuba University approved the survey protocol, including use of data from the 2012 survey.

### Participants

For the 2012 survey, participating schools were non-randomly chosen based on access to faculty members who could administer the survey at each school. Each school independently decided which student cohort years would participate in the survey. The researchers did not specify the distribution methods or target cohort years when sending a set of questionnaire to each school. The researchers were informed about whether participants were preclinical or clinical by each school.

For the 2016 survey, we sent forms requesting study participation to the Deans of all 80 medical schools in Japan from April to May 2016. Preclinical and clinical students in medical schools whose Deans approved study participation were invited. In general, medical students receive education for 6 years in Japan: They complete general liberal arts classes in the first 2 years, lectures on basic medicine in the next 2 years, and clinical practical training in the last 2 years. We asked the person in charge of administering the survey at each school to administer the survey to both preclinical and clinical students, if possible. We asked them to select a cohort year for preclinical students, according to feasibility, among students who had not yet begun their clinical clerkship programs at the time of the survey. We specified that first to third year students were favorable. Clinical students in Japan are in their fifth and sixth years of medical school. The 11-month investigation period ran from the beginning of a school year to the end of a school year. Thus, the inclusion of fifth-year students in the study would have meant that some participants had just started their clinical training, whereas others had experienced clinical training for almost 1 year. We assumed that including students with more clinical training would better demonstrate the impact of clinical training on the degree of exposure to the pharmaceutical industry. Therefore, we included only sixth-year clinical students, as these students had all experienced one year of clinical training. We confirmed the cohort years of preclinical students and the sizes of the selected classes before sending a set of questionnaires to each school.

### Survey instrument

The cover page of the questionnaire stated the purpose of the study and confirmed the voluntary nature of participation, as well as the confidentiality of responses. No incentives were offered for participation. The survey questions asked whether participants had ever accepted one or more of four items (stationery, a medical textbook, a brochure, or a lunch), and some additional questions that were asked in the 2012 survey were also included [16]. The questionnaire was not pretested for validity and reliability before being administered in the study.

### Survey administration

From May 2016 to March 2017, we sent a set of questionnaires to a staff or faculty member at each participating school, as determined by that institution’s Dean. Paper questionnaires were distributed and filled out with pens in a class with required attendance and collected on the same occasion. Completed questionnaires were returned by mail.

### Analysis

Descriptive statistics were computed for all variables. We used Pearson chi-square tests to compare the responses on accepting the four items in the 2012 and 2016 surveys. Two-tailed *p*-values less than .05 were considered to indicate statistical significance. SPSS, Version 23.0 was used for all statistical analyses.

## Results

### Participant characteristics

In the 2012 survey, 43 of the 80 medical schools in Japan participated. A total of 24 schools were national, 5 were prefectural, and 14 were private. Forty medical schools (50%) participated in the 2016 study. Of these, 22 were national, 6 were prefectural, and 12 were private. Twenty-two schools participated in the survey in both 2012 and 2016. Of the 9132 students in the surveyed classes, there were 6771 evaluable responses, making the overall response rate 74.1%, which exceeded the 60% generally considered necessary for a survey to be valid. The response rate was 72.8% (3395/4661) among preclinical students and 75.5% (3376/4471) among clinical students. Cronbach’s alpha was 0.83 for the 2012 survey and 0.85 for the 2016 survey. Table 1 shows the demographic characteristics of the survey participants in both 2012 and 2016. In the 2012 survey, where the cohort years of participating students were decided at each school according to feasibility, there were no first- to third-year preclinical students, two-thirds of the participants were in their fourth year, and the remaining one-third were in their fifth year. In the 2016 survey, first-, second-, and third-year students accounted for 21%, 32%, and 44% of the preclinical students, respectively. Regarding clinical students, there were approximately equal numbers of fifth- and sixth-year students in the 2012 survey; in the 2016 survey, clinical students were defined as being in their sixth year.

**Table 1:** Characteristics of respondents.

	Pre-clinical		Clinical	
	2012	2016	2012	2016
	n = 2660	n = 3395	n = 2672	n = 3376
	n (%)	n (%)	n (%)	n (%)
Year of study				
1st	0 (0)	705 (20.8)	0 (0)	0 (0)
2nd	0 (0)	1101 (32.4)	0 (0)	0 (0)
3rd	0 (0)	1479 (43.6)	0 (0)	0 (0)
4th	1755 (66.0)	110 (3.2)	0 (0)	0 (0)
5th	853 (32.1)	0 (0)	1369 (51.2)	0 (0)
6th	53 (2.0)	0 (0)	1303 (48.8)	3376 (100)
Ownership of schools				
National	1496 (56.2)	1756 (51.7)	1728 (64.7)	1819 (53.9)
Prefectural	326 (12.3)	438 (12.9)	173 (6.5)	364 (10.8)
Private	838 (31.5)	1201 (35.4)	771 (28.9)	1193 (35.3)

### Comparison between 2012 and 2016

In both 2012 and 2016, one in three preclinical students reported having accepted stationery from the pharmaceutical industry (36.8% and 30.4%, respectively; *p* < .001). More preclinical students in 2012 than in 2016 reported having accepted a brochure (31.2% vs. 17.6%, respectively; *p* < .001) or a lunch (21.2% vs. 13.0%, respectively; *p* < .001), whereas fewer preclinical students reported having accepted a textbook in 2012 than in 2016 (4.1% vs. 5.3%, respectively; *p* = .03).

Comparing the clinical students’ responses in 2012 and 2016, 97.1% and 98.3% (*p* = .001) had accepted stationery, 97.4% and 98.6% (*p* = .001) had accepted a brochure, and 96.6% and 98.8% (*p* = .001) had accepted a lunch, respectively. Ten percent more students had received a textbook in 2016 than in 2012 (26.7% vs. 16.9%, respectively; *p* < .001) (Table 2).

**Table 2:** Interaction between medical students and the pharmaceutical industry ‡*.

	Pre-clinical					Clinical				
	2012 (n = 2660)		2016 (n = 3395)		Comparison of proportions, 2012 versus 2016†	2012 (n = 2672)		2016 (n = 3376)		Comparison of proportions, 2012 versus 2016†
Type of gift or event	n	%	N	%	*p value*	n	%	n	%	*p value*
Stationery	980	36.8	1031	30.4	< .001	2594	97.1	3318	98.3	0.001
Medical textbook	110	4.1	181	5.3	0.03	452	16.9	901	26.7	< .001
Brochure	831	31.2	598	17.6	< .001	2602	97.4	3328	98.6	0.001
Lunch provided at a promotional presentation	563	21.2	440	13.0	< .001	2581	96.6	3332	98.8	< .001

^‡^ Sample sizes varied by item because of nonresponse. The percentages of nonresponse were less than 0.18%.

* Percentages for all items were significantly higher among clinical students than among preclinical students (chi-square test, *p* < .001).

^†^ Chi-square test

## Discussion

This was a follow-up to a study conducted in 2012, which evaluated Japanese medical students’ interactions with the pharmaceutical industry. Most of the clinical students reported having accepted stationery, a brochure, and a lunch in both 2012 and 2016. The higher rate at which clinical students interacted with the pharmaceutical industry, compared with preclinical students, was similar to the pattern seen among Japanese physicians in a previous survey in 2008, in which 96% of respondents reported receiving stationery [17].

The degree of interaction between clinical students and the pharmaceutical industry increased slightly but significantly from 2012 to 2016. In particular, approximately 10% more students reported having accepted a textbook in 2016 than in 2012. This increase was observed despite a 2008 recommendation by the Association of American Medical Colleges being translated into Japanese in 2012 and the JPMA’s 2013 release of the revised Code of Practice [15], which was expected to restrict medical students’ and physicians’ interactions with the industry. One year after the 2008 revision of the ethics codes of academic societies in the United States, there was a decrease in the number of student interactions with the pharmaceutical industry [5]. A follow-up survey of the relationships between physicians and the industry in the United States showed a reduction in their interactions from 2004 to 2009, despite the lack of regulations banning gifts and meetings with pharmaceutical representatives [18]. The authors of this previous study considered the reasons for this decrease to be increased attention to the propriety of physician–industry interactions by the press and professional organizations, increased public reporting of these interactions, new institutional policies, and company cutbacks on marketing expenses. The current survey was administered 3 years after the revision of the promotion code by the JPMA, which set an upper limit on gifts. It seems natural that the interaction was not found to decrease, because the monetary values of the types of interaction examined in the present survey were below the upper limit set by the code. However, we would expect the code to change the marketing strategies of the industry, as well as institutional policies, which could decrease the interaction [18]. Actually, however, our findings indicate that the latest revision of the code did not decrease the interaction between clinical students and the pharmaceutical industry, and other regulation such as self-regulation by professional societies is probably required to decrease medical students’ interaction with the industry. Although preclinical students’ interactions with the pharmaceutical industry did decrease, this may have been because of a greater number of lower-year participants in 2016 than in 2012.

This study had several limitations. First, only half of all medical schools in Japan participated in the study, raising concerns about participation bias. Many schools declined to participate because of the survey content, and others may have had negative viewpoints regarding educating students about the relationship with the pharmaceutical industry. Second, the recruitment method differed between 2016 and 2012: In 2012, schools were non-randomly chosen based on access to faculty members who could administer the survey at each school. Thus, a simple comparison between the 2012 and 2016 results may not reflect actual changes. Third, the respondents may have reported socially desirable responses despite the provision of anonymity and the survey being self-administered; this might have led to an underestimation of their industry interactions.

In the future, it is expected that professional societies will formulate guidelines on relationships between physicians or medical students and the pharmaceutical industry. Interactions between medical students and the pharmaceutical industry need to be closely monitored after regulations come into effect.

In conclusion, this study revealed that students’ interactions with the pharmaceutical industry barely changed between the 2012 and 2016 surveys, and these interactions were not influenced by the revised promotion code. Discussions about the regulation of the relationship with the pharmaceutical industry are required in the field of medical education.

## Supporting information

S1 Table Survey questionnaire in English and in Japanese. (DOCX)

## References

[1] Sierles FS, Brodkey AC, Cleary LM, Mccurdy FA, Mintz M, Frank J, et al Medical students’ exposure to and attitudes about drug company interactions: a national survey. JAMA. 2005;294(9):1034–42. 10.1001/jama.294.9.1034 16145023

[2] Austad KE, Avorn J, Kesselheim AS. Medical students’ exposure to and attitudes about the pharmaceutical industry: a systematic review. PLoS Med. 2011;8(5):e1001037 10.1371/journal.pmed.1001037 21629685 PMC3101205

[3] Lieb K, Koch C. Medical students’ attitudes to and contact with the pharmaceutical industry: a survey at eight German university hospitals. Dtsch Arztebl Int. 2013;110(35–36):584–90. 10.3238/arztebl.2013.0584 24078838 PMC3785017

[4] Etain B, Guittet L, Weiss N, Gajdos V, Katsahian S. Attitudes of medical students towards conflict of interest: A national survey in France. PLoS One. 2014;9(3):e92858 10.1371/journal.pone.0092858 24671179 PMC3966819

[5] Sierles FS, Kessler KH, Mintz M, Beck G, Starr S, Lynn DJ, et al Changes in medical students’ exposure to and attitudes about drug company interactions from 2003 to 2012: a multi-institutional follow-up survey. Acad Med. 2015;90(8):1137–46. 10.1097/ACM.0000000000000686 25785675

[6] Spurling GK, Mansfield PR, Montgomery BD, Lexchin J, Doust J, Othman N, et al Information from pharmaceutical companies and the quality, quantity, and cost of physicians’ prescribing: a systematic review. PLoS Med. 2010;7(10):e1000352 10.1371/journal.pmed.1000352 20976098 PMC2957394

[7] DeJong C, Aguilar T, Tseng C-W, Lin GA, Boscardin WJ, Dudley RA. Pharmaceutical industry–sponsored meals and physician prescribing patterns for medicare beneficiaries. JAMA Intern Med. 2016;176(8):1114–22. 10.1001/jamainternmed.2016.2765 27322350

[8] AAMC. Industry Funding of Medical Education: Report of an AAMC Task Force. 2008. http://www.ohsu.edu/xd/about/services/integrity/coi/gifts/upload/AAMCindustryfunding.pdf (Accessed October 3, 2018)

[9] Carlat DJ, Fagrelius T, Ramachandran R, Ross JS, Bergh S. The updated AMSA scorecard of conflict-of-interest policies: a survey of U.S. medical schools. BMC Med Educ. 2016;16(1):202 10.1186/s12909-016-0725-y 27519253 PMC4983088

[10] Grande D. Limiting the influence of pharmaceutical industry gifts on physicians: self-regulation or government intervention? J Gen Intern Med. 2010;25(1):79–83. 10.1007/s11606-009-1016-7 19756874 PMC2811591

[11] Agrawal S, Brennan N, Budetti P. The Sunshine Act-effects on physicians. N Engl J Med. 2013;368(22):2054–7. 10.1056/NEJMp1303523 23718163

[12] Vuorenkoski L, Valta M, Helve O. Effect of legislative changes in drug promotion on medical students: questionnaire survey. Med Educ. 2008;42(12):1172–7. 10.1111/j.1365-2923.2008.03169.x 19120947

[13] Steinbrook R. Controlling conflict of interest—proposals from the Institute of Medicine. N Engl J Med. 2009;360(21):2160–3. 10.1056/NEJMp0810200 19403898

[14] PhRMA. Code on Interactions with Healthcare Professionals. 2009. http://www.phrma.org/sites/default/files/pdf/phrma_marketing_code_2008.pdf (Accessed October 3, 2018)

[15] Japan Pharmaceutical Manufacturer Association. JPMA Code of Practice. 2013. http://www.jpma.or.jp/english/policies_guidelines/pdf/code_practice.pdf (Accessed October 3, 2018)

[16] Miyata Y. A national survey of medical student-pharmaceutical industry relationships. Igaku-Kyoiku. 2013;44(1):13–9. Japanese.

[17] Saito S, Mukohara K, Bito S. Japanese practicing physicians’ relationships with pharmaceutical representatives: a national survey. PLoS One. 2010;5(8): e12193 10.1371/journal.pone.0012193 20730093 PMC2921334

[18] Campbell EG, Rao SR, DesRoches CM, Iezzoni LI, Vogeli C, Bolcic-Jankovic D, et al Physician professionalism and changes in physician-industry relationships from 2004 to 2009. Arch Intern Med. 2010;170(20): 1820–6. 10.1001/archinternmed.2010.383 21059976

