# Supplementary material for: Follow-up survey of Japanese medical students’ interactions with the pharmaceutical industry
Source: PLoS One. 2018 Nov 2;13(11):e0206543. doi: 10.1371/journal.pone.0206543 (PMC6214546; doi:10.1371/journal.pone.0206543)
Supplement: S1 Table — (DOCX) [file pone.0206543.s001.docx]

Original version

問：医学部在学中に以下のことを経験したことがありますか。（各項目、ひとつに○）

1. 製薬企業からボールペンやメモ帳などの文房具を受け取ったことがありますか。

| 1. ある | 2. ない |
| --- | --- |

1. 製薬企業から医学の教科書や本来有料の診療ガイドラインなどを無償で受け取ったことがありますか。

| 1. ある | 2. ない |
| --- | --- |

1. 製薬企業から製品説明パンフレットを受け取ったことがありますか。

| 1. ある | 2. ない |
| --- | --- |

1. 製薬企業の製品説明会で提供された弁当を食べたことがありますか。

| 1. ある | 2. ない |
| --- | --- |

Question: Have you experienced the following while in medical school? (Circle one answer for each question)

English version

1. Have you ever accepted stationery such as pens and notepads from a pharmaceutical company?
2. Yes 2. No
3. Have you ever accepted a medical textbook or a book of clinical practice guidelines from a pharmaceutical company?
4. Yes 2. No
5. Have you ever accepted a brochure about a pharmaceutical company’s products?
6. Yes 2. No
7. Have you ever had a lunch provided at a promotional meeting about a company’s products?
8. Yes 2. No
